# IMPACT OF ENVIRONMENTAL SATISFACTION ON SLEEP QUALITY AMONG SOUTH ASIAN CAREGIVERS FOR OLDER ADULTS

**DOI:** 10.1093/geroni/igae098.3121

**Published:** 2024-12-31

**Authors:** Srujana Chekuri, Aziza Siddiqui, Dinesh Karki, Julie Blaskewicz Boron

**Affiliations:** University of Nebraska Omaha, Omaha, Nebraska, United States; University of Nebraska Omaha, Omaha, Nebraska, United States; University of Nebraska Omaha, Omaha, Nebraska, United States; University of Nebraska Omaha, Omaha, Nebraska, United States

## Abstract

Caregivers for older adults rely heavily on good sleep quality for their general health and ability to provide care. This study investigated the impact of environmental satisfaction, both at home and in the surrounding neighborhood, on sleep quality among South Asian caregivers for older adults, specifically those not living with the care recipients. Participants completed a survey focused on South Asian caregivers’ sleep quality and satisfaction with their surroundings, such as the cleanliness of living spaces, noise levels, and the condition of neighborhood walkways and cycling lanes. A series of regression analyses were used to examine relationships. Results demonstrated a statistically significant relationship between environmental satisfaction and sleep quality. Specifically, high satisfaction with home cleanliness, and low noise levels were positively associated (p< 0.05) with increased sleep duration. Similarly, the state of outdoor infrastructure, such as well-kept sidewalks, and cycling routes were also positively associated (p< 0.05) with more hours of sleep among caregivers. This highlights the significant role of favorable indoor and outdoor environments in enhancing sleep quality among South Asian caregivers for older adults, pointing to the potential benefits of targeted environmental interventions. These findings further emphasize the need of a supportive environment for the well-being of South Asian caregivers, even when they are not living with their care recipient. The focus on a specific cultural group may limit generalizability. Thus, future studies should incorporate diverse caregiver samples to explore the generalizability of results across different cultural contexts and living situations (e.g., residing with or without the care recipient).

